# Bagaza Virus in Himalayan Monal Pheasants, South Africa, 2016–2017

**DOI:** 10.3201/eid2512.190756

**Published:** 2019-12

**Authors:** Jumari Steyn, Elizabeth M. Botha, Carina Lourens, Jacobus A.W. Coetzer, Marietjie Venter

**Affiliations:** University of Pretoria, Pretoria, South Africa

**Keywords:** Bagaza virus, Flavivirus, Himalayan monal pheasant, viruses, South Africa, zoonoses

## Abstract

Bagaza virus (BAGV) has not been reported in birds in South Africa since 1978. We used phylogenetic analysis and electron microscopy to identify BAGV as the likely etiology in neurologic disease and death in Himalayan monal pheasants in Pretoria, South Africa. Our results suggest circulation of BAGV in South Africa.

The flavivirus genus of family *Flaviviridae* consists of 53 virus species, including arboviruses of medical and veterinary relevance, such as West Nile virus and Bagaza virus (BAGV). BAGV was isolated in 1966 from *Culex* mosquitoes in the Bagaza district of Central African Republic (*1*). In 1978, BAGV was isolated from turkeys with clinical signs similar to Israel turkey meningoencephalitis virus (ITV) in South Africa (*2*). BAGV infection causes neurologic disease in avian species, especially turkeys and other members of the *Phasianidae* family; 1 report suggests that BAGV and ITV are the same viral species (*3*). 

BAGV also has been detected in various mosquito species in western Africa (*4*,*5*), India (*6*), and the Arabian Peninsula (*7*) and in wild partridges in Spain (*8*). No evidence of the virus has been reported in other parts of Africa. Zoonotic transmission was reported in India after patients with acute encephalitis demonstrated 15% positivity for BAGV neutralizing antibodies (*6*). We report detection of BAGV in fatalities in Himalayan monal pheasants in South Africa during 2016–2017.

## The Study

In April 2016, two Himalayan monal pheasants (*Lophophorus impejanus*) and 1 tragopan pheasant (*Tragopan melanocephalus*) suddenly died on a property northeast of Pretoria, Gauteng Province, South Africa. In June 2017, this property had another 4 monal pheasants that displayed signs of lethargy and ataxia and died within a day. Around the same time, a residence in the northern suburbs of Pretoria had 5 monal and 2 tragopan pheasants that exhibited neurologic signs and died. That residence had another incidence in 2018 when a monal pheasant exhibited neurologic disease. Also in 2018, a monal pheasant was found dead in North West Province, South Africa. The cause of these deaths was unknown. All the birds were adults that were locally bred from parents imported from Belgium >2 years before.

Brain tissue from the 16 birds was sent to the Department of Veterinary Tropical Diseases (DVTD), University of Pretoria (Pretoria, South Africa), for virus isolation and to the Centre for Viral Zoonoses (CVZ), University of Pretoria, for zoonotic arbovirus investigations. At the CVZ, we extracted RNA from the brain tissues by using the RNeasy Mini Kit (QIAGEN, https://www.qiagen.com) according to manufacturer’s instructions under Biosafety Level 3 conditions. We used nested real-time reverse transcription Pan-Flavi assay targeting the nonstructural coding gene 5 (NS5) (*9*) to identify the etiologic agent (*10*,*11*). To obtain a larger NS5 gene segment, we performed additional PCR using SuperScript III/Platinum Taq Mix (Invitrogen, https://www.thermofisher.com) and the MAMD (*9*) and FLAVI-2 (*10*) primers with the following cycling conditions: 50°C for 30 min; 94°C for 15 min; 35 cycles of 94°C for 45 s, 50°C for 45 s, 72°C for 1 min; and 72°C for 10 min. We successfully obtained a larger NS5 gene segment for phylogenetic analyses, but only for 4 positive birds.

We assembled and edited sequence data by using CLC Main WorkBench (https://www.qiagenbioinformatics.com) and performed multiple sequence alignments using the online version of MAFFT (http://mafft.cbrc.jp/alignment/server/index.html) with default parameters. We used MEGA 6.06 (https://www.megasoftware.net) to view, edit, and truncate the datasets. We downloaded reference sequences for the flavivirus genus from GenBank (*12*). We conducted maximum likelihood analysis in RAxML (*13*), invoking the autoMRE bootstopping function applying a general time-reversible plus gamma model with default 4 rate categories on both datasets. We performed an analysis on the longer dataset by using BEAST version 1.8 (http://beast.community) and a relaxed log-normal clock, general time-reversible plus gamma model, and default priors to generate a maximum clade credibility tree (MCC). We ran a Markov chain Monte Carlo analysis for 10^6^ generations, saving every 1,000th tree. We estimated effective sample size by using Tracer version 1.6 (http://tree.bio.ed.ac.uk/software/tracer) with an effective sample size value >200. We used TreeAnnotator version 1.8 (http://beast.community) to generate the MCC tree and discarded 15% as burn-in. We displayed bootstrap and posterior probabilities on RAxML topology.

We performed virus isolation on all PCR-positive samples. We inoculated brain tissue supernatant onto a confluent monolayer of baby hamster kidney fibroblast cells (BHK-21 line) in 25 cm^2^ tissue culture flasks and incubated at 37°C for 1 h. Then we added Dulbecco’s Minimum Essential Medium (ThermoFisher Scientific, https://www.thermofisher.com) containing 2% fetal bovine serum and 0.1 mg/mL gentamycin. We harvested cells and supernatant when 80% of the cell monolayer showed cytopathic effect and sent these to the Electron Microscopy Unit of The University of Pretoria and to the CVZ for molecular identification.

**Figure 1:**
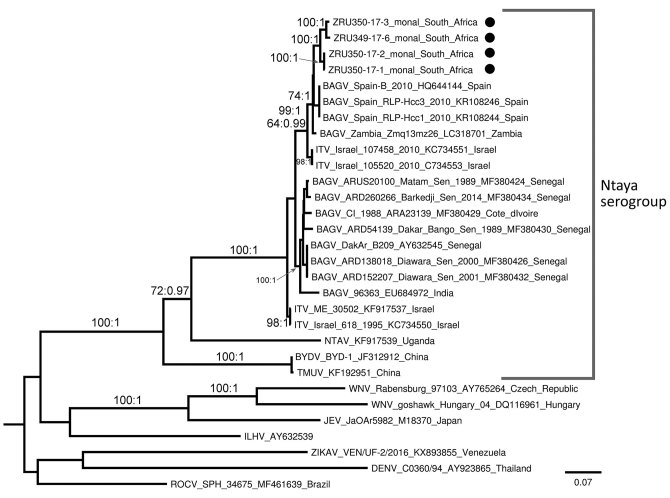
Maximum-likelihood phylogram of BAGV isolated in samples from Himalayan monal pheasants (black dots), South Africa, 2016–2017. Phylogram represents partial (1,079 nt) nonstructural coding gene 5 (NS5; taxa = 30). Bootstrap support with values >60 indicated on branches with posterior probabilities >0.95 from a maximum clade credibility tree. BAGV strains from this study are available in GenBank under the following accession nos.: ZRU349/17/6, no. MN329586; ZRU350/17/1, no. MN329584; ZRU350/17/2, no. MN329585; ZRU350/17/3, no. MN329587. Scale bar indicates nucleotide substitutions per site. BAGV, Bagaza virus; BYDV, Baiyangdian virus; DENV, Dengue virus; ILHV, Ilheus virus; ITV, Israel turkey meningoencephalitis virus; JEV, Japanese encephalitis virus; NTAV, Ntaya virus; ROCV, Rocio virus; TMUV, Tembusu virus; WNV, West Nile virus; ZIKAV, Zika virus.

The Pan-Flavi assay targeting the NS5 gene resulted in amplicons of the expected size in 8/13 (61.5%, 95% CI 35.1%–88.0%) Himalayan monal pheasants but not in the 3 tragopan pheasants tested. Neurologic signs were reported before death in 7/8 (87.5%, 95% CI 0.5%–66.5%) positive birds, but 1/8 (12.5%, 95% CI 0–2.1%) was found dead and its clinical signs are unknown. Inqaba Biotec (https://www.inqababiotec.co.za) performed Sanger sequencing; we confirmed all positive samples as BAGV using phylogenetic analysis of the flavivirus genus NS5 PCR regions at CVZ. Phylogenetic analyses were based on partial nucleotide sequences of NS5 from genomic positions 9091–9280 (166 nt) and 9030–10109 (1,079 nt) and were used to compare the identified strains with other flaviviruses. Analyses confirmed the molecular results from all 8 Himalayan monals as BAGV in the Ntaya virus group with a bootstrap value of 92, sister to BAGV strains from Spain (bootstrap value 68) (Appendix Figure). The 4 strains for which we amplified a larger region (1,079 nt) formed 2 well-supported sister groups, both with a bootstrap value of 100 and phylogenetic probability of 1, with nucleotide similarities of 97.7%–99.7%, and highest nucleotide identity (96.7%–97.7%) to strain Zambia_Zmq13mz26 (GenBank accession no. LC318701.1) isolated from a mosquito (Figure 1).

**Figure 2:**
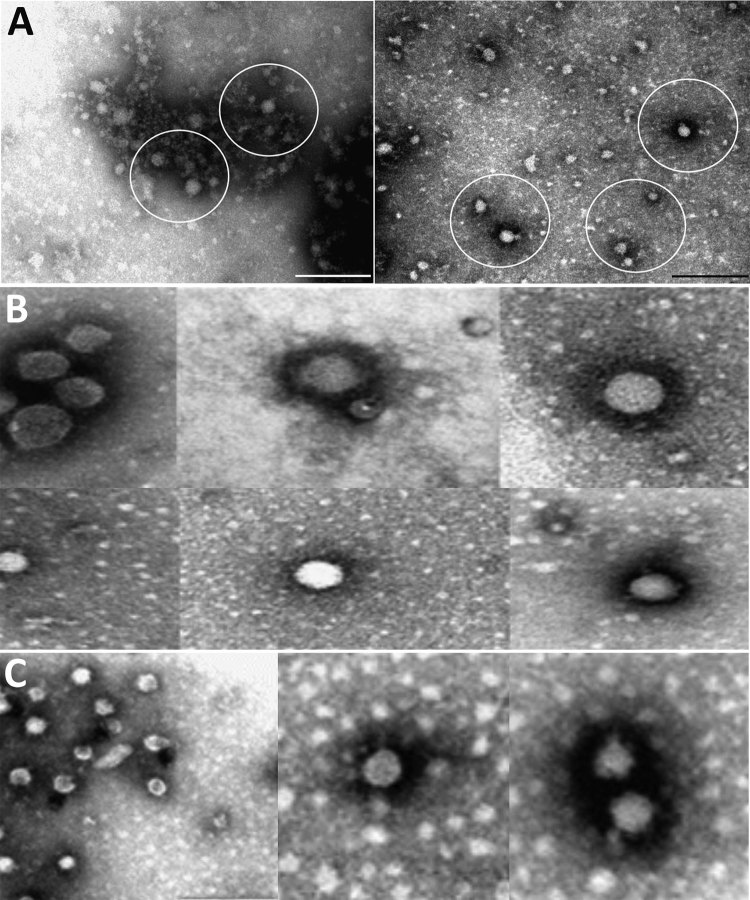
Electron microscopy of Bagaza virus isolated in samples from Himalayan monal pheasants, South Africa, 2016–2017. A) Circles indicate occasional particles with size range and approximate morphology of *Flaviviridae* observed in samples ZRU350_17_1 and ZRU350_17_2. Scale bars indicate 200 nm. B) A few isolated fringed isometric particles of 40–65 nm (top row) and free-lying smooth-surfaced particles of 25–40 nm (bottom row) of suspected *Flaviviridae* observed in sample ZRU349_17_6. C) A few free-lying smooth-surfaced particle cores of 30–40 nm (C1) and a cluster of fringed isometric particles of 40–50 nm (C2–3) of suspected *Flaviviridae* observed in sample ZRU349_17_6.

Electron microscopy on 3 BAGV cultures (sample nos. ZRU350_17_1, ZRU350_17_2, and ZRU349_17_6) (Appendix Table) from 2017 confirmed the presence of *Flaviviridae* particles (Figure 2). We observed fringed isometric and free-lying smooth-surfaced particles typical of *Flaviviridae* (Figure 2, panels B and C).

## Conclusions

We detected BAGV in the offspring of monal pheasants imported from Belgium to South Africa. We sequenced BAGV strains and found they monophyletically clustered with strains from Spain rather than strains from West Africa. However, nucleotide similarities in the large gene segment were highest when compared with a strain from Zambia that was isolated from a *Cx. quinquefasciatus* mosquito (GenBank accession no. LC318701.1; *14*), an endemic species in South Africa that could be a BAGV vector. We noted 2 distinct monophyletic clusters of BAGV, a cluster composed of strains from West Africa and older strains and a cluster containing the newly sequenced birds with BAGV from Spain and more recent strains that could indicate several circulating strains or genotypes. 

We used virus isolation and electron microscopy results to confirm the etiology of the agent as a flavivirus. The causative link between the clinical symptoms of the monal pheasants and evidence of BAGV infection should be regarded with caution because we did not exclude other possible infectious and noninfectious etiologies. However, detection of BAGV in the brain suggests crossing of the blood–brain barrier and exclusion of other flaviviruses, arboviruses, and orthobunyaviruses suggests BAGV as a probable cause. Future work will focus on next-generation sequencing to obtain full genomes because initial attempts were unsuccessful. More data are needed to determine the endemicity of BAGV and the reservoir host and vectors of BAGV in South Africa and to define the seroprevelance of these infections in birds and possibly in humans.

Appendix Additional information on detection of Bagaza virus in Himalayan monal pheasants, South Africa, 2016–2017.
