# Supplementary material for: Bagaza Virus in Himalayan Monal Pheasants, South Africa, 2016–2017
Source: Emerg Infect Dis. 2019 Dec;25(12):2299–302. doi: 10.3201/eid2512.190756 (PMC6874265; doi:10.3201/eid2512.190756)
Supplement: Appendix — Additional information on detection of Bagaza virus in Himalayan monal pheasants, South Africa, 2016–2017. [file 19-0756-Techapp-s1.pdf]

Article DOI: https://doi.org/10.3201/eid2512.190756

Bagaza Virus in Himalayan Monal Pheasants, South Africa, 2016–2017

Appendix

Appendix Table. Summary of Bagaza virus cultures used for electron microscopy, using cell line BHK (BSR), in investigation of Bagaza virus in Himalayan monal pheasants (*Lophophorus impejanus*), Pretoria, South Africa, 2017

Sample no.	Passage no.	BHK (BSR) passage no.
ZRU350_17_1	3	47
ZRU350_17_2	3	73
ZRU349_17_6	4	35

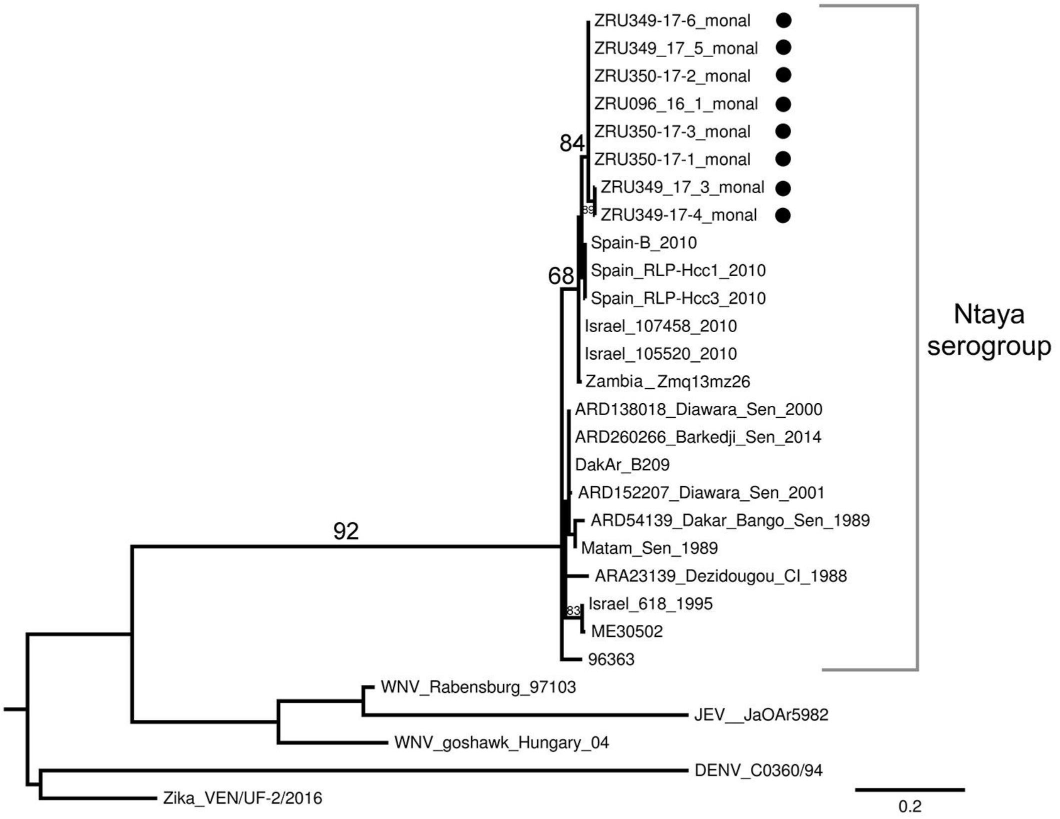

Appendix Figure. Maximum likelihood phylogram of Bagaza virus isolated in samples from Himalayan monal pheasants, South Africa, 2016–2017. Phylogram represents partial (166 nt) nonstructural coding gene 5 (NS5; taxa = 29). Bootstrap support with values of >60 indicated on branches. Black circles indicate strains sequenced in this study. Scale bar indicates nucleotide substitutions per site.
